# Cognitive Ability and Self-Control’s Influence on High School Students’ Comprehensive Academic Performance

**DOI:** 10.3389/fpsyg.2021.783673

**Published:** 2021-12-10

**Authors:** Yueqi Shi, Shaowei Qu

**Affiliations:** School of Humanities and Social Sciences, University of Science and Technology Beijing, Beijing, China

**Keywords:** cognitive ability, self-control, comprehensive academic performance, hierarchical linear model, interactive influence

## Abstract

This study uses a hierarchical linear model (HLM) to examine the effects of cognitive ability and self-control on comprehensive academic performance among students in a high school in Beijing. The study included 572 participating students, including 291 boys and 281 girls, ranging in age from 16 to 18 years old. In this study, the individual level of students’ cognitive abilities are used as the first-level variables, including memory ability (MA), information processing ability (IPA), representation ability (RA), logical reasoning ability (LRA), and thinking transformation ability (TCA). Consider self-control at the class level as the second-level variable. The research results show that the five cognitive abilities have a significant positive impact on comprehensive academic performance. Self-control plays an active role in regulating the relationship between RA, LRA, TCA, and comprehensive academic performance.

## Introduction

Zhao (2017) believes that academic performance refers to the performance of students’ learning effects after a period of learning of knowledge and skills. Under the current education system, schools often express students’ academic performance in the form of examinations. Liu (2019) believes that academic performance, especially college entrance examination scores, affects students’ future development, and clarifying the factors affecting academic performance will undoubtedly be of great help to each student’s study. Yang (2020) believes that academic performance, as a test of students’ learning results, reflects students’ mastery of the knowledge they have learned, and is a key indicator to measure students’ learning. Li (2019) believes that academic performance is the academic knowledge and skills acquired after learning and training, and it is a concentrated expression of students’ learning status and level. Under the current education system in China, academic performance is a measure of a student’s learning achievements and important parameters for further studies. Therefore, academic performance is an important indicator for evaluating the learning effects of students.

Cognitive ability and self-control are always important factors affecting students’ academic performance (Liang et al., 2020), especially since the adoption of online teaching in the post-pandemic era. Self-control has become a key factor affecting students’ academic performance (Schulz, 2021), but it is not clear how cognitive ability and self-control together affect the comprehensive academic performance of students. This research takes high school students’ individual-level cognitive abilities and class-level self-control abilities as the research variables, and explores the mechanism of their influence on comprehensive academic performance and the impact of their interaction on comprehensive academic performance.

### The Impact of Cognitive Ability on Academic Performance

Cognitive ability has an important restrictive effect on whether young people can achieve academic success. Cognitive ability refers to the ability of the human brain to process, store, and extract information, including attention, memory, and reasoning ability. It is a key psychological element for people to successfully complete an activity (Sternberg and Sternberg, 2009). Cognitive ability is currently one of the most researched and stable predictors of academic performance (Stadler et al., 2016). Previous studies have focused on the direct impact of individual-level cognitive ability on academic performance (Kuncel et al., 2004; Miriam et al., 2011). In a study of 4,749 junior high school students by Xu and Li (2015), it was found that selective attention, short-term memory, and reasoning ability are significant predictors of language and mathematics performance. Reasoning ability directly affects academic performance, while selective attention and short-term temporal memory indirectly affect academic performance through reasoning ability. Rohde and Thompson (2007) found that cognitive ability directly predicts academic performance, and the correlation is as high as 0.38. Cognitive ability and academic performance in language and mathematics have a moderate to high degree of correlation, which can explain the larger component of variation in academic performance (Deary et al., 2006; Rohde and Thompson, 2007; Plomin, 2011). Deary et al. (2006) conducted a 5-year follow-up study of more than 70,000 British children and found that the correlation between general cognitive ability at 11 years of age and academic performance at 16 years of age was 0.81. The strongest predictive ability can explain 58.6 and 48% of the variation in the dependent variable, respectively. These research findings also support the information processing theory (Deary et al., 2006; Rohde and Thompson, 2007; Xu and Li, 2015), which states that the higher the students’ cognitive ability, the faster and more accurately they can pay attention to key information, efficient memory coding, and the output of more effective information, thus leading to higher academic performance (Liu and Wang, 2000; Zhang and Zhang, 2011). In contrast, if the overall level of cognitive ability is low or a certain cognitive ability is lacking, part of the information will be lost in the process of information processing, reducing the output of effective information, and leading to lower academic performance (Miriam et al., 2011). Chen (2016) believes that cognitive abilities have an impact on high school students’ chances and choices for further studies. Students with higher cognitive abilities are more likely to attend regular high schools, and conversely, students with lower cognitive abilities are more likely to attend vocational schools. Based on previous research results, cognitive ability often plays a significant role in the achievement of higher academic performance.

Although the relationship between general cognitive ability and academic performance can better reflect the closeness of the two, it is difficult to deeply reflect the inner relationship between them. In fact, “in the learning context, cognitive ability is very important in human learning activities, which can be reflected in a deeper level by including specific cognitive abilities in the scope of the investigation,” (David, 2005) because learning activities not only involve different specific abilities, but are also related to how these different abilities work together. Throughout different studies, there is much controversy over the influence model of cognitive ability on academic performance (Formazin et al., 2011). Zhang (2008) found that the correlation coefficients between logical reasoning ability (LRA), Chinese language, and Mathematics scores were all around 0.3, while the thinking transformation ability (TCA) was not significantly correlated with the scores of the two subjects. However, Xu and Li (2015) found that thinking TCA has a significant correlation with performance in the two subjects, and the correlation coefficient between representation ability (RA) and the performance of Chinese and Mathematics is between 0.4 and 0.5. These results confirm that it is difficult to comprehensively and systematically reveal the complex interaction of individual cognitive factors on academic performance when only examining the impact of a single dimension of cognitive ability on a single academic performance. Moreover, previous studies tended to study the influencing factors of cognitive ability on a single subject, and there is a lack of research on the influencing factors of cognitive ability on the comprehensive performance of multiple subjects.

Therefore, this study combines the classification of cognitive ability by Wo (2000); Xu and Li (2015), and Liang et al. (2020), these three researchers have conducted a lot of research and exploration on the five cognitive abilities of working memory, information processing, logical reasoning, representation, and thinking transformation in the article, and obtained scientific and effective research conclusions, and summarized different types of recognition. The test method of knowledge ability. In particular, Wo has developed a software system for measuring these five cognitive abilities. The number of students measured in mainland China has exceeded 2 million, and he has obtained normative data applicable to local students in mainland China. Reliability and validity have been fully tested, and can accurately measure the cognitive ability of students. In addition, Wo (2010) applied for and obtained a Chinese invention patent for the cognitive ability assessment system. According to the research of Wo (2010), this research divides the cognitive ability into memory ability (MA), information processing ability (IPA), RA, LRA, and thinking conversion ability (TCA). It explores the specific impact of different cognitive abilities on the comprehensive academic performance of the three subjects of Chinese, mathematics, and English, and put forward the following hypotheses:

Hypothesis 1a: Memory Ability (MA) in Individual Cognitive Ability of Students is positively correlated with comprehensive academic performance.
Hypothesis 1b: Information processing ability (IPA) is positively correlated with comprehensive academic performance.
Hypothesis 1c: Representation ability (RA) is positively correlated with comprehensive academic performance.
Hypothesis 1d: Logical reasoning ability (LRA) is positively correlated with comprehensive academic performance.
Hypothesis 1e: Thinking conversion ability (TCA) is positively correlated with comprehensive academic performance.

### The Influence of Self-Control on Academic Performance

People have emphasized the important role of cognitive ability factors in the learning process (Kuncel et al., 2004; Miriam et al., 2011; Stadler et al., 2016). In China, many scholars have conducted research on students’ academic performance and Cognitive ability, and the general public generally recognizes that students with strong Cognitive ability have good academic performance (Zhang, 2008; Xu and Li, 2015; Chen, 2016). However, a series of studies have shown that intelligence is only one of the factors that determine students’ academic performance (Shao, 1983). An individual’s academic performance is not only determined by one aspect of his or her state, but is also affected by his or her overall positive mental state.

Self-control refers to the ability of individuals to consciously restrict and manage their own cognition, emotions and behaviors in accordance with social standards or their own wishes without external supervision and external restrictions (Xie, 2009). Duckworth and Seligman (2010) and other scholars have studied the influence of discipline on academic performance, Self-control has a positive effect on the performance of eighth-grade students in the United States. Dai (2013) conducted related research on the relationship between self-control, emotional stability, and academic performance in junior high school students and found that there was a significant positive correlation between the self-control of high school students and their academic performance. Zhao (2017) and others conducted research on the relationship between self-control and academic performance in upper primary school students and found that self-control can significantly predict academic performance. Wang (2003) used a self-compiled self-control questionnaire to study the relationship between the self-control ability and academic performance of middle school students, and surveyed 885 students from the first to second year in Shanghai. The results show that the self-control ability and academic performance of middle school students are Significantly positive correlation. Wang (2004) did a research on the relationship between self-control and academic performance of Mongolian and Han junior high school students. The study surveyed 622 junior high school students. The results showed that there is a significant positive correlation between the self-control ability of junior high school students and academic performance. Previous studies have shown that students’ self-control is related to academic performance. However, the mechanisms of the influence of self-control on the academic performance of high school students is still unclear. Existing research rarely study the influence of the two dimensions of cognitive ability and self-control on academic performance at the same time. Based on the shortcomings of existing research, this research will establish a more comprehensive model to study the impact of high school students’ cognitive ability and self-control on academic performance. Taking into account the nested structure of the data, a hierarchical linear model (HLM) was selected for analysis. Based on this, the following hypotheses were proposed:

Hypothesis 2a: Self-control moderates the relationship between Memory Ability (MA) and comprehensive academic performance, such that the relationship is stronger for higher levels of self-control.
Hypothesis 2b: Self-control moderates the relationship between Information processing ability (IPA) and comprehensive academic performance, such that the relationship is stronger for higher levels of self-control.
Hypothesis 2c: Self-control moderates the relationship between Representation ability (RA) and comprehensive academic performance, such that the relationship is stronger for higher levels of self-control.
Hypothesis 2d: Self-control moderates the relationship between Logical reasoning ability (LRA) and comprehensive academic performance, such that the relationship is stronger for higher levels of self-control.
Hypothesis 2e: Self-control moderates the relationship between Thinking conversion ability (TCA) and comprehensive academic performance, such that the relationship is stronger for higher levels of self-control.

This study selected students from a high school in Beijing as the participants. The academic performance of the students in this school is at a low level in the Beijing area. Many students have a poor learning attitude and are very irresponsible for their studies. Therefore, self-control ability will greatly affect students’ academic performance.

**FIGURE 1:**
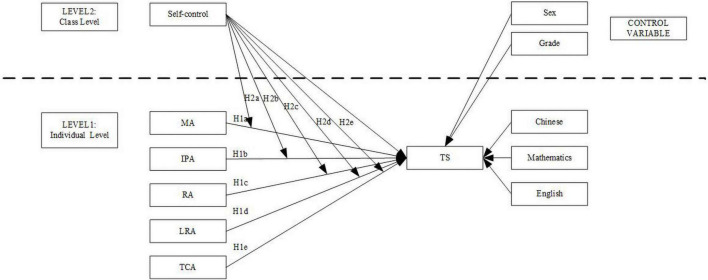
Hypothesized model. MA, Memory ability; IPA, Information processing ability, RA, Representation ability; LRA, Logical reasoning ability, TCA, Thinking conversion ability. TS–Comprehensive Academic performance.

The main research variables and the research relationship are shown in Figure 1.

## Materials and Methods

### Participants

This study selects 572 students from 50 classes in a high school in Beijing as a sample, with 10–13 students in each class. All students selected in the sample must go to school as normal and have not gone through suspension. At the same time, all students must have been studying in this school since they were in high school, and cannot include transfer students from other regions or schools. Among them, with 291 boys (50.87%) and 281 girls (49.23%), and the age range is 16–18 years old. Among the students, there were 225 in the first year (115 boys and 110 girls), 178 in the second year (83 boys and 95 girls) and 169 in the third year (93 boys and 76 girls). The study included 572 participating students, 291 boys and 281 girls, ranging in age from 16 to 18 years old.

### Procedure

All the tests in this study were conducted on the campus of the Affiliated Middle School of the University of Science and Technology, Beijing. The school teachers uniformly organized all students to enter the computer lab for testing. The test content included high school students’ cognitive ability and self-control tests. The overall duration of the test was 2 h and 30 min. This research was approved by the Research Ethics Committee of the School of Humanities and Social Sciences, University of Science and Technology, Beijing, we obtained written informed consent from all students and their parents before the test, and the data used in the study were provided by the Affiliated Middle School of the University.

### Measures

#### Cognitive Ability

The cognitive ability test uses the stimulus information cognitive ability value test system developed by Wo (2010).

The Stimulus information cognitive ability value test system used the world’s leading EEG ultraslow fluctuation analyzer and ASL504/501 eye trackers as basic research methods, started from the brain mechanisms of individual psychological development, combined laboratory experiments and field experiments, adopted subtraction reaction time and addition reaction time (accurate to the nanosecond), microgenetics and other technologies, used a form of microcomputer manual operation that greatly improves the discrimination and accuracy of test results, and used the accumulated sample norms of more than 2,000,000 people to make the subjects’ personal quantitative indicators comparable to people of the same age.

The test questions of IPA include three parts: image selection reaction, graph comparison reaction, and image matching reaction. The test questions of memory ability are two parts: digital forward, reverse memory, and image matching reaction. The test questions of RA include three parts: spatial image manipulation, spatial image reasoning, and spatial image comparison. The test subject of thinking TCA is a text-picture matching test. The test questions of LRA are conceptual reasoning and logical law reasoning.

The cognitive index analysis method provides the subject with visual, auditory, and/or tactile stimulation information through a stimulation information-providing device to obtain feedback information generated by the subject according to the stimulation information. The feedback information is collected and/or received by the information input device and the information collection device and then sent to the analysis and processing device for analysis so that the analysis and processing device obtains random feedback information subconsciously generated when the subject receives the stimulus information collected by the information collection device. The first analysis data were used to assist in correcting the second analysis data obtained from the inherent feedback information directly put in by the subject through the information input device so that the analysis and processing device determined the subject’s performance based on the first analysis data and the second analysis data. A standard score model (Z-score model) was established based on the current cognitive index value for the subject and the average index value for the group. We calculated the Z score of each index item for the subject. In the same way, average response accuracy was calculated based on the data in the response accuracy database. A Z-score model was established based on the current subject’s response accuracy and the average value, and the subject’s response accuracy Z-score was calculated. Finally, the abovementioned cognitive index value Z score and reaction accuracy Z score were added to obtain a comprehensive Z score. The comprehensive Z score is converted into a T score that characterizes the subject’s cognitive ability. The T score is the quantitative measurement of cognitive ability. The cognitive ability values obtained by this test method are centered at 100 and have a normal distribution trend in the range of ± 50, which has high discrimination validity. Cronbach’s alpha in this study ranged from 0.80 to 0.90.

#### Self-Control

The Self-Control test scale for middle school students was designed by Zhang et al. (2005). We set up six questions for the test, including a reverse question. The Positive problem was evaluated on a 5-point Likert scale: 1 (Very inconsistent), 2 (relatively inconsistent), 3 (uncertain), 4 (Relatively consistent), and 5 (Very consistent)—this test will test the score of each item. The test set up reverse questions (R), and the reverse questions are set to 5 (Very inconsistent), 4 (relatively inconsistent), 3 (uncertain), 2 (Relatively consistent), and 1 (Very consistent). For example, the scores of each item for self-control are recorded as A1, A2, A3, A4, A5, and A6, where A4 is the reverse-scored question. Then, the total score for self-control is A = (A1+A2+A3+A4+A5+A6)/6. Then the calculated total score is converted into a Z score based on the mean and standard deviation, and then the Z score is converted into a T score with an average of 50 and a standard deviation of 10, then the T score is the test student’s self-control ability value. The Cronbach α coefficient of each dimension of the scale is between 0.60 and 0.93, and the test-retest reliability is 0.85. The validity is 0.91.

#### Comprehensive Academic Performance

This research takes students’ recent test scores as their comprehensive academic performance. Because students choose different subjects, this research only selects the compulsory subjects of Chinese, Mathematics, and English for all students. In order to reduce the impact of different levels of difficulty of examination questions in different grades on the research results, the student scores used in this study are obtained based on the percentage of students’ rankings. The calculation formula is: score = (total number of students-student ranking)/total number of students*100. The score range is 0–100. The total scores of the three subjects were accumulated.

### Data Analysis

The data of this study involves two dimensions of students’ individual level of cognitive ability and class level of self-control, and there is a level of interaction. The multilevel data structure can be used in the traditional multiple linear regression model. For example, the basic assumption of the ordinary least squares (OLS) method is linear and positive. However, it is obvious that multi-level data cannot satisfy the independence of the same variance and random error terms, which reduces the accuracy of the model results. Compared with OLS, HLM is more suitable for processing multi-layer structure data, decomposing the original single random error to the corresponding levels, which can greatly improve the effect of model fitting (David, 1997); at the same time, decomposition of the total variation of the dependent variable into different levels of variation, systematic analysis of the influence of different levels of variables on the dependent variable, and cross-level interactions are performed in order to obtain more accurate estimation results.

In this study, the cognitive ability of students at the individual level is regarded as the first level of the hierarchical linear regression model, which includes five variables; the self-control at the class level is the second level, which includes one variable. SPSS 19.0 and HLM software were used for data analysis.

The progression of the HLM analysis is as follows: first, do not bring any level of variables into the zero-model test to determine whether the data is suitable for HLM analysis; second, bring in the first level of cognitive ability variables (random coefficient model), and analyze the impact of cognitive ability on academics; third, only the second-level self-control variables (intercept model) are introduced to analyze the individual effect of self-control on comprehensive academic performance. Finally, the first-level cognitive ability and the second-level self-control abilities are brought into the regression equation (full model) to analyze the impact of the interaction between cognitive abilities and self-control on comprehensive academic performance (Goldstein, 2010).

## Results

### Descriptive and Bivariate Analyses

The mean values, standard deviations, and intercorrelations of the variables are presented in Table 1. The five cognitive abilities were significantly correlated with comprehensive academic performance (TS): thus, Hypothesis 1a-1e were supported. The self-control showed a significant negative correlation with TS.

**TABLE 1:** Means, standard deviations, and intercorrelations for variables.

	M	SD	1	2	3	4	5	6	7
1.MA	104.85	15.91	–						
2.IPA	102.25	13.32	0.156**	–					
3.RA	106.20	7.31	–0.033	0.205**	–				
4.LRA	103.16	9.99	0.039	0.074	0.084*	–			
5.TCA	94.09	17.40	0.171**	0.400**	0.338**	0.074	–		
6.SELF-CONTROL	106.31	15.68	0.311**	0.455**	0.264**	0.225**	0.531**	–	
7.TS	160.50	58.08	0.551**	0.647**	0.432**	0.350**	0.755**	0.667**	–

N = 572. *p < 0.05; **p < 0.01.

### Hierarchical Multiple Regression Analysis

#### Zero Model

Before performing HLM, it is necessary to determine whether this method is suitable for use. Generally, it is necessary to test the consistency within a group and the differences between groups. In a HLM, a zero-model analysis must be performed first. The zero-model does not contain any predictor variables and is mainly used to test whether it is necessary to use a multilevel model for analysis and to calculate the influence of high-level factors on the dependent variable. See the Supplementary Material for the formula.

The dependent variable *TS_ij_* represents the total score for student i; β_0*j*_ is the intercept, which represents the average score of class j students; γ_*ij*_ represents the variation of class j student i around the average score of class j (individual-level random effect), and the variance is σ^2^; γ_00_ is the mean of all students’ performance; *u*_0*j*_ represents the variation in various mean values around the overall mean (random effect at the regional level); and the variance is τ_00_. In this way, the variation in the dependent variable is decomposed into intragroup and intergroup variations. By calculating the proportion of intergroup variation in the total variation, the magnitude of the influence of the regional level on the dependent variable can be obtained.

In the zero-model, the most important value is the interclass correlation coefficient ICC (1) (intraclass correlation), ICC (1) = τ_00_ /(σ^2^ + τ_00_). In addition, Keith (1988) believes that when ICC (1) is greater than 0.138, it is considered to be a high correlation. By calculating ICC (1) = 0.333 > 0.138 in this study, it was found that 33.3% of the variance in students’ comprehensive academic performance was caused by the differing self-control of students. The general linear regression model is not suitable for a unified analysis of the results of this study. It is necessary to use the HLM model in order to analyze the inconsistency of the differences between the groups. ICC (2) characterizes the degree of difference between the dependent variables in different groups, indicating the reliability of the model. In this study, ICC (2) = 0.821 > 0.70 (James, 1982), indicating that students’ comprehensive academic performance is in the different self-control groups of students. Since there are significant differences between the two, it is suitable to use the HLM model for analysis. The results of the analysis are presented in Table 2.

**TABLE 2:** Zero model regression results.

Fixed effect	Coefficient	S.E.	df	*T-*ratio	*p*
Intercept	162.795609	5.486905	49	29.670	0.000
Random effect	S.E.	Variance	df	Chi-square value	*p*
*u*_0_	35.51330	1261.19434	49	249.33555	0.000
γ_*ij*_	50.30352	2530.44419			

At the same time, because the student’s self-control values used in this study are derived from the questionnaire, where each index value is aggregated from six questions, the aggregation degree of the index should be verified before the analysis. In order to verify that the data have homogeneity within a group and the degree of interrater agreement between different evaluators, the calculated Rwg_*j*_ = 0.864 > 0.7 in this study (James et al., 1984), which shows that the deviation between the Rwg_*j*_ value of students’ self-control and the mean value is small, the index has a good degree of aggregation, and the aggregation index formed by each item is reasonable.

#### Full Model

The values of cognitive ability, self-control, and interaction items are brought into the model, and grade and gender are used as control variables to analyze the influence of cognitive ability and self-control on comprehensive academic performance. See the Supplementary Material for the formula.

Here, γ_31_ and γ_71_ are the slopes at which the learning attitude of level-2 intercepts the slopes of the respective variables in the first level. In the complete model, the fixed effects include the total intercept, base cognitive ability, self-control, and the interaction of these two levels. The random effect, *u*_0*j*_, also represents the randomness of unobserved or unobservable self-control. The effect, *u*_3*j*_ -*u*_7*j*_, represents the part of the influence of cognitive ability on the dependent variable that varies with self-control and cannot be explained by the group variable included in the model. The results of the analysis are presented in Table 3.

**TABLE 3:** Hierarchical linear model regression results.

Fixed effect		Full model	
		Coefficient	S.E.
	Intercept γ_00_	1150.717414*** (0.000)	2.287450
Control variable	GRADE (γ_20_)	5.625206*** (0.000)	0.540922
	SEX (γ_10_)	0.633757 (0.385)	0.728651
Cognitive ability	MA (γ_30_)	1.553573*** (0.000)	0.030501
	IPA (γ_40_)	1.479794*** (0.000)	0.027549
	RA (γ_50_)	1.553179*** (0.000)	0.059689
	LRA (γ_60_)	1.520598*** (0.000)	0.048074
	TCA (γ_70_)	1.493005*** (0.000)	0.031347
Self-control	Self-control (γ_01_)	2.485198*** (0.000)	0.127194
Interaction layer	Self-control*MA (γ_31_)	0.02890 (0.302)	0.02766
	Self-control*IPA (γ_41_)	0.03989* (0.083)	0.02259
	Self-control*RA (γ_51_)	0.09022* (0.073)	0.04934
	Self-control*LRA (γ_61_)	0.02510 (0.490)	0.03606
	Self-control*TCA (γ_71_)	0.3752** (0.037)	0.02483

*P < 0.1; **P < 0.05; ***P < 0.001.

In the case of grade and gender as the control variables, MA, IPA, RA, LRA, and TCA have a significant positive impact on comprehensive academic performance (TS) (*P* < 0.001), indicating that Hypotheses 1a, 1b, 1c, 1d, and 1e are supported.

Self-control has a significant positive effect on comprehensive academic performance (TS) (γ_01_ = 2.485,*P* < 0.001). However, the grade has a significant effect on students’ comprehensive academic performance (γ_20_ = 6.256, *P* < 0.001), indicating that the relationship between self-control and students’ comprehensive academic performance is affected by the age of students.

Self-control plays a positive regulatory role in the relationship between IPA and comprehensive academic performance (TS) (γ_41_ = 0.04, *P* < 0.1), indicating that Hypothesis 2b is supported. Self-control regulates the relationship between RA and comprehensive academic performance (TS) (γ_51_ = 0.09, *P* < 0.1), indicating that Hypothesis 2c is supported. Self-control also plays a positive role in TCA and comprehensive academic performance (TS) (γ_71_ = 0.735, *P* < 0.05); thus, Hypothesis 2e is also valid.

## Discussion

### Impact of Cognitive Ability on Comprehensive Academic Performance

Previous studies have shown that cognitive ability is a psychological feature and a psychological condition for the normal learning activities (Stadler et al., 2016). In China, elementary and junior high schools belong to the state’s compulsory education stage. The school effect of students is not tested or evaluated, and the development of students’ learning ability is not paid much attention to. At the high school stage, because students have to take the college entrance examination to enter the university, both teachers and students pay attention to improving academic performance, and also attach importance to the development of students’ learning ability. Therefore, the high school stage is a good time for students to effectively improve their learning abilities. Different subjects have different ability requirements for students (Zuo and Wang, 2016; Liang et al., 2020).

MA (memory ability) can effectively help students improve memory, recitation, and other supporting content (Liang et al., 2020). At the same time, it interacts with IPA to improve students’ reading comprehension ability. This is particularly evident in Chinese and English reading. Therefore, in students with good MA, academic performance is also better (Yu et al., 2014). RA (representational ability) plays an important role in the learning of spatial knowledge in subjects such as mathematics (Shao et al., 2004). At the same time, RA can also stimulate associative memory to recite Chinese and English related knowledge, which makes students perform better academically (Lin et al., 2003). TCA (thinking conversion ability) is reflected in the speed and accuracy of thought transformation, so any subject learning is inseparable from this ability (Zeng and Xie, 2021). Especially in high school mathematics, students with strong TCA can easily summarize and master ideas and methods of completing new math problems, and proficiently apply them to similar problems, and achieve good comprehensive academic performance (Liu, 1988). LRA is divided into two types: inductive reasoning and deductive reasoning (Zhang, 2008). The influence of LRA on academic performance is mostly concentrated in mathematics (Zhu et al., 2020). In recent years, the examination of students in Chinese and English has also been emphasized with the changes in the content of Chinese examinations. Given the logic and rigor of Chinese and English exams (Hu, 2017), LRA has also been shown to affect the scores in the reading part of the Chinese and English exams. IPA is mainly represented by reading comprehension ability and is also related to the efficiency of listening to lectures (Hu et al., 2010). Higher IPA fosters in students a greater ability to understand and master reading in the classroom, the formation of knowledge systems, and better academic performance in examinations (Yuan and Wen, 2003).

At the same time, it was found that the five cognitive abilities have an impact coefficient of approximately 1.5, that is, each individual ability value increases. Each increase of 1 unit will increase the corresponding comprehensive academic performance by 1.5 points, indicating the balance of the students’ examination subjects at this stage. The examination questions focused on the examination of students’ comprehensive abilities rather than the investigation of a single factor.

### The Influence of Self-Control on Comprehensive Academic Performance

Self-control is a behavior habit, with self-management as its essence. It refers to the behavioral ability that students can use to force themselves to complete the expected goals that should be accomplished based on certain principles and norms, relying on their own conscience and willpower without supervision (Liu, 2020).

The self-control ability of high school students means that they have higher concentration and the ability to resist external temptations, which can help high school students not to be influenced by the outside world. For high school students, as long as they improve their self-control ability, they can think independently, complete homework, study and reduce time wasting, thereby increasing their learning efficiency and improving their academic performance quickly and continuously.

According to Beijing’s teaching characteristics, the school ends at 16:30 in the afternoon, while the remaining time is at the discretion of the students. Students with strong self-control can master the time well and spend a lot of time on learning activities to consolidate their knowledge and improve their studies. Thus, students with high self-control can often invest more time in subject learning and achieve better comprehensive academic performance.

At the same time, compared with cognitive ability, it was found that self-control has a significant and positive influence on comprehensive academic performance. For every additional unit of a student’s self-control, their comprehensive academic performance will increase by 2.49 points, which is more effective than any single learning ability. This research shows that the key factor restricting the improvement of students’ comprehensive academic performance is self-control, not the student’s cognitive ability.

At the same time, in terms of the influence of the control variables of grade and gender, gender had no significant effect on comprehensive academic performance, whereas the effect of grade on comprehensive academic performance is very significant. Each increase in grade increases corresponding comprehensive academic performance by 5.6 points, indicating that with the increase in students’ age, students’ adaptability and learning methods continue to mature. With the increase of age, students’ self-control ability has also been improved (the average self-control ability of the first grade is 102, and the average self-control ability of the third grade is 109). Students in the upper grades can conduct better self-management, are less likely to experience emotional breakdown, and can calmly analyze the reasons and deal with them well when faced with substantial progress or regression in performance (Zheng and Zhang, 2021). At the same time, the emotional state of senior students is relatively stable, and their emotional regulation ability will be higher, and they can better control themselves from being affected by other things, thereby improving learning efficiency and improving comprehensive academic performance.

### The Impact of the Interaction Between Cognitive Ability and Self-Control on Comprehensive Academic Performance

Regarding the interaction between cognitive ability and self-control, it was found that self-control has a positive moderating effect on the influence of IPA, RA, and TCA on comprehensive academic performance. The higher the self-control, higher is a student’s degree of concentration; thus, when students go to read and study, the advantages of IPA and presentation ability can be fully utilized to improve the speed of understanding and mastery, thereby improving learning efficiency and comprehensive academic performance (Wang, 2011). Consequently, students with high self-control will invest a lot of time in learning, so that through a lot of exercises, they can exercise the flexibility and divergence of learning thinking, and give play to the advantages of TCA, which will help them in the learning process. Students can continue to sort out, summarize, and master the laws of learning, doing so more efficiently (Wo and Lin, 2000), and improving their comprehensive academic performance.

In addition, it can be seen that the moderating effect of self-control in TCA and comprehensive academic performance is far greater than that of IPA and presentation ability. This is because with the progress of China’s examination reform, the importance of examination questions in the assessment of students’ thinking has become increasingly important, exceeding the assessment of the mastery of basic knowledge (Lin, 2021).

In addition, MA, LRA, and self-control ability have no significant influence on comprehensive academic performance. This is because MA is a kind of innate cognitive ability possessed by students, which does not change with students’ behavior. Therefore, for students with high MA and poor self-control ability, students can quickly complete the memory task and achieve good academic performance without too high self-control ability. LRA is more of a cognitive ability that relies on students’ in-depth thinking to improve. The logical reasoning questions in the exam generally require students to spend a lot of time and energy to make it, and students with high self-control ability usually have time arrangements. Integrity and organization (Zheng and Zhang, 2021). They can balance the time arrangement during the examination process, and will not spend a lot of time on logical reasoning questions, so the interactive effect of LRA and self-control ability on academic performance is not significant.

### Limitations and Future Directions

All samples in this study are from a Beijing high school, and the sample size was relatively small. The next step will be to select more schools in other provinces in China for research and comparison. In addition, when considering the factors that affect students’ cognitive abilities, this study only considers self-control, and does not use more factors for analysis. The next step will be to analyze the impact of students’ planning ability, execution ability, and persistence, and the impact of the project is expected to provide more valuable research results. At the same time, this research only considered high school students in the research process, which has limitations in the age of the research group, next research will expand the age range of the research, from elementary school, junior high school, high school, and university.

## Conclusion

This study uses a HLM to study the impact of cognitive ability and self-control on students’ comprehensive academic performance. Doing so, it was found that MA, IPA, RA, LRA, and TCA have a significant positive impact on comprehensive academic performance. Self-control has a significant positive influence on comprehensive academic performance, and self-control plays a positive role in regulating IPA, RA, TCA, and comprehensive academic performance. Therefore, in the process of improving students’ comprehensive academic performance, we should not only focus on the training of cognitive ability, but also pay attention to cultivating students’ self-control ability. Teachers should set clear learning goals for students in the classroom and formulate executable self-control plan. Parents should pay attention to urging students to complete the study plan on time and create a good self-control learning environment for students’ learning. Students should also have an active sense of self-control, and cultivate good behaviors with the assistance of teachers and parents, so as to improve their overall academic performance.

## Data Availability Statement

The original contributions presented in the study are included in the article/Supplementary Material, further inquiries can be directed to the corresponding author/s.

## Ethics Statement

The studies involving human participants were reviewed and approved by the Research Ethics Committee of the School of Humanities and Social Sciences, University of Science and Technology Beijing. Written informed consent from the participants’ legal guardian/next of kin was not required to participate in this study in accordance with the national legislation and the institutional requirements.

## Author Contributions

YS and SQ contributed to conception and design of the study. YS contributed to data collection, performed the statistical analysis, and wrote the first draft of the manuscript. Both authors contributed to manuscript revision, read, and approved the submitted version.

## Conflict of Interest

The authors declare that the research was conducted in the absence of any commercial or financial relationships that could be construed as a potential conflict of interest.

## Publisher’s Note

All claims expressed in this article are solely those of the authors and do not necessarily represent those of their affiliated organizations, or those of the publisher, the editors and the reviewers. Any product that may be evaluated in this article, or claim that may be made by its manufacturer, is not guaranteed or endorsed by the publisher.
