# Supplementary material for: Cognitive Ability and Self-Control’s Influence on High School Students’ Comprehensive Academic Performance
Source: Front Psychol. 2021 Dec 10;12:783673. doi: 10.3389/fpsyg.2021.783673 (PMC8702492; doi:10.3389/fpsyg.2021.783673)
Supplement: Supplementary file 1 [file Table_2.docx]

**Formula**

(1) Zero Model

The zero-model can be expressed through the following formula:

Level-1: $TS_{ij}=\beta_{0j}+\gamma_{ij}$ （1）

Level-2: $\beta_{0j}=\gamma_{00}+u_{0j}$ （2）

Overall model: $TS_{ij}=\gamma_{00}+u_{0j}+\gamma_{ij}$ （3）

(2)Full Model:

Level-1:

$\begin{matrix} TS_{ij}=\beta_{0j}+\beta_{1j}\text{（}SEX_{ij}\text{）}+\beta_{2j}\text{（}GRADE_{ij}\text{）}+\beta_{3j}\text{（}MA_{ij}\text{-}\overline{MA}_{.j}\text{）}+\beta_{4j}\text{（}IPA_{ij}\text{-}\overline{IPA}_{.j}\text{）+}\beta_{5j}\text{（}RA_{ij}\text{-}\overline{RA}_{.j}\text{）} \\ \text{+}\beta_{6j}\text{（}LRA_{ij}\text{-}\overline{LRA}_{.j}\text{）+}\beta_{7j}\text{（}TCA_{ij}\text{-}\overline{TCA}_{.j}\text{）}+\gamma_{ij} \end{matrix}$ (4)

Level-2:

$\beta_{0j}=\gamma_{00}+\gamma_{01}*(SELFCONTROL_{j})+u_{0j}$ (5)

$$\beta_{1j}=\gamma_{10}$$

$$\beta_{2j}=\gamma_{20}$$

$$\beta_{3j}=\gamma_{30}+\gamma_{31}*(SELFCONTROL_{j}-\overline{SELFCONTROL}_{.})+u_{3j}$$

$$\beta_{4j}=\gamma_{40}+\gamma_{41}*(SELFCONTROL_{j}-\overline{SELFCONTROL}_{.})+u_{4j}$$

$$\beta_{5j}=\gamma_{50}+\gamma_{51}*(SELFCONTROL_{j}-\overline{SELFCONTROL}_{.})+u_{5j}$$

$$\beta_{6j}=\gamma_{60}+\gamma_{61}*(SELFCONTROL_{j}-\overline{SELFCONTROL}_{.})+u_{6j}$$

$$\beta_{7j}=\gamma_{70}+\gamma_{71}*(SELFCONTROL_{j}-\overline{SELFCONTROL}_{.})+u_{7j}$$

Overall model:

$\begin{matrix} TS_{ij}=\gamma_{00}+\gamma_{01}*(SELFCONTROL_{j})+\gamma_{10}*SEX_{ij}+\gamma_{20}*GRADE_{ij} \\ +\gamma_{30}*(MA_{ij}\text{-}\overline{MA}_{.j}\text{）}+\gamma_{31}*(SELFCONTROL_{j}-\overline{SELFCONTROL}_{.})*(MA_{ij}\text{-}\overline{MA}_{.j}\text{）} \\ +\gamma_{40}*\text{（}IPA_{ij}\text{-}\overline{IPA}_{.j}\text{）+}\gamma_{41}*(SELFCONTROL_{j}-\overline{SELFCONTROL}_{.})*\text{（}IPA_{ij}\text{-}\overline{IPA}_{.j}\text{）} \\ \text{+}\gamma_{50}*\text{（}RA_{ij}\text{-}\overline{RA}_{.j}\text{）+}\gamma_{51}*(SELFCONTROL_{j}-\overline{SELFCONTROL}_{.})*\text{（}RA_{ij}\text{-}\overline{RA}_{.j}\text{）} \\ \text{+}\gamma_{60}*\text{（}LRA_{ij}\text{-}\overline{LRA}_{.j}\text{）+}\gamma_{61}*(SELFCONTROL_{j}-\overline{SELFCONTROL}_{.})*\text{（}LRA_{ij}\text{-}\overline{LRA}_{.j}\text{）} \\ \text{+}\gamma_{70}*\text{（}TCA_{ij}\text{-}\overline{TCA}_{.j}\text{）+}\gamma_{71}*(SELFCONTROL_{j}-\overline{SELFCONTROL}_{.})*\text{（}TCA_{ij}\text{-}\overline{TCA}_{.j}\text{）} \\ +u_{0j}+u_{3j}*(MA_{ij}\text{-}\overline{MA}_{.j}\text{）}+u_{4j}*\text{（}IPA_{ij}\text{-}\overline{IPA}_{.j}\text{）}+u_{5j}*\text{（}RA_{ij}\text{-}\overline{RA}_{.j}\text{）}+u_{6j}*\text{（}LRA_{ij}\text{-}\overline{LRA}_{.j}\text{）}+u_{7j}*\text{（}TCA_{ij}\text{-}\overline{TCA}_{.j}\text{）}+\gamma_{ij} \end{matrix}$ (6)
